# A Laboratory-Based Study on Multiple Biomarker Testing in the Diagnosis of COVID-19-Associated Pulmonary Aspergillosis (CAPA): Real-Life Data

**DOI:** 10.3390/diagnostics13010114

**Published:** 2022-12-30

**Authors:** Cornelia Lass-Flörl, Miriam Knoll, Wilfried Posch, Michael Joannidis, Timo Mayerhöfer, Robert Breitkopf, Romuald Bellmann

**Affiliations:** 1 Institute of Hygiene and Medical Microbiology, Medical University of Innsbruck, A-6020 Innsbruck, Austria; 2 Department of Internal Medicine, Division of Intensive Care and Emergency Medicine, Medical University of Innsbruck, A-6020 Innsbruck, Austria; 3 Department of Anaesthesia and Intensive Care, Medical University of Innsbruck, A-6020 Innsbruck, Austria

**Keywords:** *Aspergillus*, CAPA, galactomannan enzyme immunoassay, *Aspergillus* PCR, conventional diagnostics in aspergillosis

## Abstract

(1) Background: Coronavirus disease 2019 (COVID-19)-associated pulmonary aspergillosis (CAPA) raises concerns to contribute to an increased mortality. The incidence of CAPA varies widely within hospitals and countries, partly because of difficulties in obtaining a reliable diagnosis. (2) Methods: Here, we assessed *Aspergillus* culture-positive and culture-negative respiratory tract specimens via direct fungal microscopy (gold standard) and compared the results with galactomannan enzyme immunoassay (GM-EIA) and *Aspergillus* PCR. (3) Results: 241 respiratory samples from patients suffering from SARS-CoV-2 pneumonia were evaluated. Results showed both diagnostic tools, *Aspergillus* PCR and GM-EIA, to be positive or negative displaying a sensitivity of 0.90, a specificity of 0.77, a negative predictive value (NPV) of 0.95, and a positive predictive value (PPV) of 0.58 in *Aspergillus* sp. culture and microscopic-positive specimens. Non-bronchoalveolar lavage (BAL) samples, obtained within a few days from the same patient, showed a high frequency of intermittent positive or negative GM-EIA or *Aspergillus* PCR results. Positivity of a single biomarker is insufficient for a proper diagnosis. A broad spectrum of *Aspergillus* species was detected. (4) Conclusions: Our study highlights the challenges of combined biomarker testing as part of diagnosing CAPA. From the results presented, we highly recommend the additional performance of direct microscopy in respiratory specimens to avoid overestimation of fungal infections by applying biomarkers.

## 1. Introduction

Invasive aspergillosis is frequently recognized in immunocompromised hosts, such as transplant recipients and patients with hematologic malignancies, patients receiving long-term or high-dose steroids, or other immunosuppressant drugs [1]. The outbreak of the current severe acute respiratory syndrome coronavirus 2 (SARS-CoV-2) pandemic led to an increase in intensive care patients with severe pulmonary disorders [2]. Thus, several reports of coronavirus disease 2019 (COVID-19)-associated pulmonary aspergillosis (CAPA) have raised concerns that this superinfection contributes to an increased mortality [2]. However, cases of CAPA were found to vary widely between hospitals and countries, and intensive care units (ICU) reported incidences from 3% to 33% [3,4]. These differences might partly be caused by difficulties in obtaining a reliable diagnosis and the lack of specific clinical presentations [4,5]; a systematic review and meta-analysis underline that the prevalence of CAPA may be exaggerated due to the use of non-standardized definitions [6].

In addition, the pandemic caused an epidemic of COVID-19-associated pulmonary mucormycosis (CAPM) in India, with more than 50,000 cases involved. Based on a Delphi consensus statement, released from the Fungal Infections Study Forum and Academy of Pulmonary Sciences in India, available data on imaging, diagnostic challenges, and the management of CAPM were summarized [7]. Most importantly, probable CAPM definition includes the demonstration of aseptate hyphae with or without growth of Mucorales in any lower respiratory tract specimens, together with compatible clinical features, risk factors, and suggestive imaging. Such a definition clearly differs from other guidelines, where culture, microscopy, and biomarkers are equated for probable definitions of invasive mold infections [8,9]. From a mycological point of view, the requirement of fungal hyphae being present in respiratory specimens for probable CAPM definitions makes sense as only hyphae correspond to hyphomycosis.

Recently, FUNgal infections Definitions in ICU patients (FUNDICU) investigators considered the visualization of fungal hyphae to be important also for probable CAPA definitions [10]. The lack of evaluation of existing definitions of culture and biomarker positivity against microscopy in CAPA cases was highlighted. A positive culture in respiratory specimens (specifically endotracheal aspirates) does not distinguish fungal colonization from infection, an issue that might be important to avoid overtreatment [11]. To close this gap, we performed a retrospective laboratory study and compared the diagnostic performance of galactomannan enzyme immunoassay (GM-EIA) and *Aspergillus* PCR with microscopy in fungal culture-positive and culture-negative respiratory tract specimens of ICU patients suffering from SARS-CoV-2 pneumonia.

## 2. Materials and Methods

The Institute of Hygiene and Medical Microbiology (HMM) of the Medical University of Innsbruck offers full-service diagnostic testing in microbiology with a main research focus on mycology; in 2018 the HMM was designated a European Confederation of Medical Mycology Excellence Centre (ECMM EC). The HMM serves amongst other Institutes for the University Hospital of Innsbruck, a tertiary care hospital covering 1900 beds including 7 intensive care units. For the lab-based analysis of fungal infections, we usually perform culture, microscopy, and/or fungal-specific PCRs, depending on the specimen available and usually only on specific request. During the 2nd COVID-19 pandemic wave, a routine screening of COVID-19 ICU patients was implemented by using an additional *Sabouraud* glucose *agar*(SAB) plate for all routinely taken respiratory specimens (37 °C for 3 days). Here, in the laboratory study (March 2021 to March 2022), leftover *Aspergillus* culture-positive respiratory tract specimens were subjected to fungal microscopy, GM-EIA (Platelia *Aspergillus* Test, Bio-Rad, Vienna, Austria), and *Aspergillus* PCR testing (MycoReal Kit *Aspergillus*, Ingenetix, Austria), to compare the diagnostic performance of biomarkers with conventional methods. In addition, culture-negative respiratory samples, from patients who previously tested culture positive, were evaluated. This study was approved by the ethical committee of the Innsbruck Medical University (EK Nr: 1150/2021, approved on 7 June 2021) and was performed according to good clinical practice.

Routine diagnostic samples from SARS-CoV-2 positive ICU patients such as bronchoalveolar lavages (BAL), bronchial-, tracheal-secretions, and sputa were aseptically divided into fractions for microscopy, *Aspergillus* PCR and GM-EIA. According to Koehler et al. [8] for GM-EIA, a single cut-off > 1.5 for BALs, single cut-off > 4.5, or twice or more cut-offs > 1.2 for non-bronchoscopic bronchial lavage samples were classified as positive. For sputum, a value of >4.5 was used [8]. Culture testing included the usage of SAB at 37 °C for 3 days, growth of *Aspergillus* species (sp.) prompted species identification via matrix-assisted laser desorption/ionization time-of-flight mass spectrometry (MALTI-TOF MS) or sequencing [12]. Microscopy was performed via calcofluor white staining (Fungi-Fluor^TM^, Polysciences, Warrington, PA, USA); mucous respiratory samples were diluted 1:10 and centrifuged for 10 min at 3000× *g*. Supernatants were applied for GM-EIA detection. Whole nucleic acids were extracted from the pellet and a single positive *Aspergillus* PCR in BAL or others was defined using a cycle threshold < 36. All data were collected by the usage of pseudo-anonymized case report forms. Statistical calculations were carried out using GraphPad Prism software, version 5.02 (GraphPad Software, https://www.graphpad.com/ (accessed on 12 October 2022)).

## 3. Results

**Figure 1:**
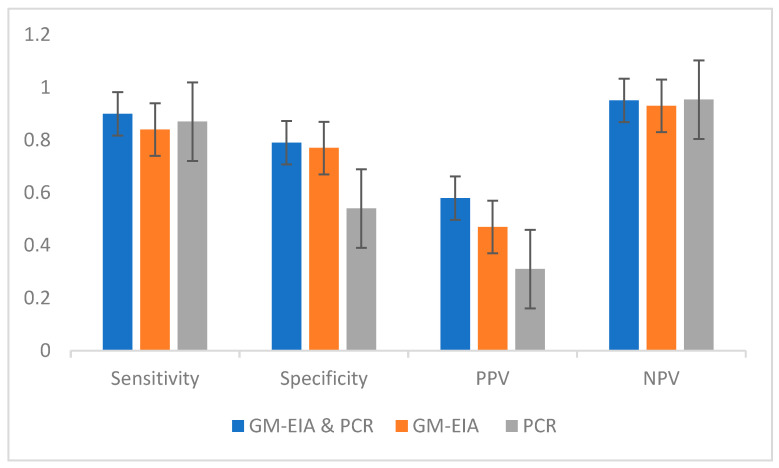
*Aspergillus* sp. culture-positive specimens (*n* = 172) analyzed via direct microscopy (gold standard), *Aspergillus* PCR, and galactomannan enzyme immunoassay testing (GM-EIA). PPV, positive predictive value; NPV, negative predictive value.

**Figure 2:**
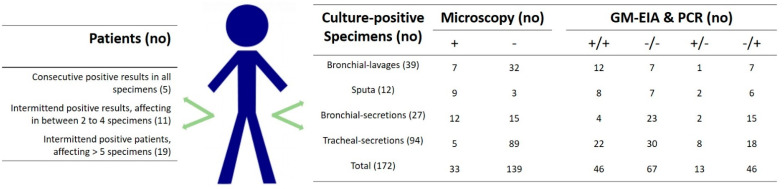
Overview of biological variance in *Aspergillus* sp. Culture-positive specimens (*n* = 172) and patients (*n* = 35).

Two hundred and forty-one various respiratory samples including BALs (*n* = 59), tracheal secretions (*n* = 123), bronchial secretions (*n* = 37), and sputa (*n* = 22) from COVID-19 ICU patients (*n* = 35) were investigated. Thereof, 172 specimens were *Aspergillus* sp. culture positive and 69 subsequent samples culture negative. We assessed *Aspergillus* PCR and GM-EIA and compared results with fungal microscopy (*Aspergillus*-like hyphae), see Table 1 and Table 2, Figure 1 and Figure 2. Results showed both diagnostic tools, *Aspergillus*-PCR and GM-EIA, to be positive or negative displaying a sensitivity of 0.90, a specificity of 0.77, a negative predictive value (NPV) of 0.95, and a positive predictive value (PPV) of 0.58 in *Aspergillus* sp. Culture-positive specimens. Similar data were obtained in culture-negative samples, see Table 2. Discordant results were observed in 79 respiratory specimens, as either GM-EIA or *Aspergillus* PCR were positive or negative, see Table 1 and Table 2. Assuming only one biomarker, GM-EIA or *Aspergillus* PCR, to be positive resulted in a sensitivity of 0.84 or 0.87, specificity of 0.77 or 0.54, NPV of 0.95 each, and PPV of 0.47 or 0.31 in *Aspergillus* sp. Culture-positive specimens. For the analysis of the *Aspergillus* culture-negative cohort, it is important to stress that these specimens were obtained during or after antifungal treatment regimens (e.g., voriconazole or isavuconazole); GM-EIA or *Aspergillus* PCR positivity led to a specificity of 0.87 and an NPV of 100; assuming only one biomarker, GM-EIA or *Aspergillus* PCR to be positive resulted in a sensitivity of 0.5 each, specificity of 0.70 or 0.83, an NPV of 0.95 each, and a PPV of 0.09 or 0.15, respectively.

Overall, positive GM optical density (OD) values ranged from 1.2–24.4, with a mean of 6.3; there were no major differences in the ODs detected between BAL and non-BAL obtained specimens (*p* = 0.3), or fungal hyphae being present. In addition, no correlation was present between positive GM and positive PCR results in non-BAL specimens. Furthermore, non-BAL samples, obtained within a few days from the same patient, showed a high frequency of intermittent positive or negative GM-EIA or *Aspergillus* PCR results, see Figure 2. In general, a high frequency of *Candida* culture-positive samples was obtained; yeasts were detected in 106 respiratory specimens. Culture-positive and *Aspergillus* PCR-positive samples included *Aspergillus fumigatus* species complex (s.c), *Aspergillus terreus* s.c, *Aspergillus flavus* s.c, *Aspergillus nidulans* s.c, and other rare representatives as well as various combinations, see Table 3. One Mucor was detected, but this isolate was not taken into account as neither GM-EIA nor the *Aspergillus*-specific PCR detects Mucorales.

## 4. Discussion

The goal of this study was to assess the value of multiple biomarker testing for the diagnosis of fungal infections in COVID-19 ICU patients, in particular, to provide evidence-based data to the clinician for the targeted use of GM-EIA, *Aspergillus* PCR, and conventional tools. The lack of validation of *Aspergillus* biomarker tests on respiratory sample types, the equation of culture, microscopy, or biomarkers (GM-EIA and PCR) for fungal disease definitions in different patient populations, prompted us to perform this lab-based study. The frequent detection of *Aspergillus* sp. or GM-EIA in airway samples from critically ill COVID-19 patients and reports of patients with CAPA who survived without receiving antifungal therapy highlight the complexity of the management [5]. As the mortality of CAPA is reported to be around 50%, antifungal therapy may, thus, be implemented early on [13]. Here, the gold standard was fungal-positive direct microscopy (*Aspergillus*-like hyphae); results showed both diagnostic tools, *Aspergillus* PCR and GM-EIA, to be positive or negative displaying a sensitivity of 0.90, a specificity of 0.77, an NPV of 0.95, and a PPV of 0.58 in *Aspergillus* sp. Culture-positive specimens. A large proportion of our specimens showed inconsistent results within GM-EIA and *Aspergillus* PCR, see Table 1 and Table 2. Irregularities were not only observed between the evaluated biomarkers, but also within the patients tested, see Figure 1. Even the proof of hyphae in non-BALs varied between specimens taken of a singular patient within a short timeframe; such biological variance underlines the huge challenges in diagnosing CAPA. Initiation of antifungal treatment based on a single positive *Aspergillus* PCR or BAL GM-EIA value may exaggerate CAPA cases and, thus, may lead to unnecessary treatment. Bormann et al. [14] came to similar discrepant results when applying serum biomarkers of 61 CAPA patients from which multiple sample types were available. Based on their study, the authors conclude that conventional mycological examination (microscopy and culture) of respiratory secretions is mandatory for a proper diagnosis. The difficulties lay in the fact of missing CAPA definitions based on the value of lower respiratory tract specimens and an equality of microcopy, serology, and culture in general (mycological evidence). Hence, the inability to classify CAPA patients is mainly due to the absence of host factors, non-typical lesions on computed tomography, and reliance on single positive lower respiratory specimens other than BALs [15]. *Aspergillus* exists as conidia (airborne) and hyphae (vegetative growth). Both phenotypes may result in a positive culture, but only vegetative growth supports disease progression; the transition from conidia to hyphae is the base of pathophysiology and, thus, in the onset and progression of a fungal disease [15]. It is therefore obvious that direct microscopy is mandatory for dealing with an evidence-based diagnosis in mycosis [16]. This recommendation is in agreement with our findings; a concordance of 85.6% was calculated for fungal microscopy and consistent results of both biomarkers. Delliere et al. [17] suggested lowering or at least specifying the quantitative cycle threshold for each specific qPCR; in their study, a threshold of 32 was associated with an increased mortality.

Among *A. fumigatus* s.c. and *A. terreus* s.c, we identified *A. flavus* s.c, *A. niger* s.c., and other rare representatives being potentially involved in CAPA; the latter species are rather untypical for invasive cases in our region, and hence may represent colonization rather than infection [1]. Particularly, culture was more often positive than *Aspergillus* PCR; this is an interesting fact, but we need to stress that out of 172 investigated culture-positive samples, only 33 displayed fungal hyphae. Our data are in accordance with the fact, that respiratory tract specimens are not sterile. Hence, vigilance is required in the interpretation of a positive culture.

The consensus CAPA case definition published by the European Confederation for Medical Mycology (ECMM) and the International Society for Human and Animal Mycology (ISHAM), categorizes patients as proven, probable, and possible CAPA [8]. Patients diagnosed through non-BAL specimens are classified as possible CAPA cases, highlighting the uncertainty of fungal diagnosis. With this in mind, the recently published Indian CAPM guidelines, for which the existence of hyphae is mandatory for probable definition (independent of fungal growth), are straightforward and support an evidence-based diagnosis [7]. The definitions by Blot et al. [18] to distinguish between putative invasive pulmonary aspergillosis and *Aspergillus* colonization in the ICU are based on a positive culture, histopathology, patient factors, and abnormal imaging. Positive histology displays the proof of an infection but assumes the investigation of tissue; biomarkers are not included as mycological criteria [19]. Using these stringent criteria, the incidence of putative invasive aspergillosis was low, and significantly lower in patients with SARS-CoV-2 pneumonia than in those with influenza pneumonia.

The 241 respiratory samples (culture negative and culture positive) investigated via multiple biomarker testing underscore modest results for PPV but high NPVs, a fact, which should be taken into account for future considerations. The relatively high frequency of false-positive GM-EIA results obtained by upper respiratory tract samples is yet unknown, but could be multiple. In a recent study, we identified *Candida* colonization as a risk factor for false-positive biomarker results [20]; BAL fluid samples from critically ill patients shared a rate of 29% false-positive GM-EIA results. Although the underlying pathomechanisms are not clear, *Candida* species quantities of ≥10^4^/mL and *Candida glabrata* were significantly associated with positive GM-EIA results. Overall, the rate of positivity was higher in GM-EIA testing than in *Aspergillus* PCR—this is somewhat wondering, as conidia are ubiquitous and it was assumed that *Aspergillus* PCR applied will significantly improve the detection rates, specifically of non-BAL guided specimens. Another explanation for inconsistent results of multiple biomarker testing may be the damage of pulmonary lung epithelium in COVID-19-associated infections [21]. It is widely recognized that biomarker testing may be influenced by a broad array of factors [22].

Our study has several limitations, starting with defining microscopy as the gold standard. It is well known that microscopy in BAL examinations is less sensitive in hematological patients. Hence, a low sensitivity considered by itself might negatively impact our study data. However, *Aspergillus* colonization in ICU patients is reported to be 36.9% [11]. Hence, we wonder whether a low sensitivity of microscopy is an issue in this patient population. Secondly, the absence of validated GM-EIA cut-off values for upper respiratory tract specimens may support the overestimation of CAPA. The performance of microscopy needs well-trained staff as the differentiation between *Aspergillus*-like hyphae with yeasts and pseudohyphae being present is difficult and may result in an inaccurate recording. The retrospective nature of our study may have had an impact on lab analysis as frozen-thawed specimens were assessed. In addition, a correlation between clinical data and patient outcome is lacking, and furthermore, we focused only on culture-positive patients; however, CAPA patients may suffer from fungal diseases without displaying culture-positive specimens.

## 5. Conclusions

In conclusion, our study highlights the challenges of combined biomarker testing as part of diagnosing CAPA. Results from non-BAL samples were poorly repeatable, and one positive biomarker seems to be insufficient for a proper diagnosis. Cut-off values for non-BAL guided techniques or even specifically for ICU-CAPA populations are highly needed; a testing algorithm to diagnose CAPA requires further studies. From the results presented here, we highly recommend the performance of direct microscopy as a standard of care—also in endotracheal aspirates to improve diagnosis. An increased awareness of potential pitfalls in using biomarkers in ICU-CAPA patients seems to be necessary.

## Figures and Tables

**Table 1:** *Aspergillus* sp. culture-positive specimens (*n* = 172) analyzed via direct microscopy (gold standard), *Aspergillus* PCR, and galactomannan enzyme immunoassay testing (GM-EIA).

Tests	Results	No. of Specimens and Microscopy		Sen	CI	Spec	CI	PPV	CI	NPV	CI	LR+	CI	LR−	CI
		+	−												
GM-EIA/PCR	+/+	27	19	0.9	0.84–0.95	0.77	0.72–0.81	0.58	0.51–0.66	0.95	0.92–0.98	3.9	3.16–4.88	0.12	0.07–0.22
	−/−	3	64												
GM-EIA/PCR	+/−	1	12	These results fall out of the analysis illustrated above, as are either positive or negative in GM-EIA or *Aspergillus* PCR.											
GM-EIA/PCR	−/+	2	44												
Single GM-EIA	+	28	31	0.84	0.78–0.91	0.77	0.74–0.81	0.47	0.40–0.54	0.95	0.93–0.97	3.8	3.17–4.55	0.19	0.12–0.29
Single GM-EIA	−	5	108												
Single PCR	+	29	63	0.87	0.81–0.93	0.54	0.50–0.59	0.31	0.26–0.36	0.95	0.92–0.97	1.93	1.72–2.18	0.22	0.13–0.36
Single PCR	−	4	76												

PCR, polymerase chain reaction; GM-EIA, galactomannan enzyme immunoassay; Sen, sensitivity; Spec, specificity; CI, Confidence interval, lower to upper CI; LR+, likelihood ratio positive; LR−, likelihood ratio negative; −, negative; +, positive.

**Table 2:** *Aspergillus* sp. culture-negative specimens (*n* = 69) analyzed via direct microscopy (gold standard), *Aspergillus* PCR, and galactomannan enzyme immunoassay testing (GM-EIA).

GM-EIA & PCR	No. (%) of Specimens and Microscopic Examination		Sensitivity	Specificity	Positive Predictive Value	Negative Predictive Value
	+	−				
+/+	0	7 (10.1)	NA	0.80	NA	100
−/−	0	42 (60.8)				
+/−	2 (2.8)	12 (17.3)	All specimens investigated showed a high frequency of yeasts detected by culture. *C. albicans* and *C. glabrata* were the dominant species involved.			
−/+	2 (2.8)	4 (4.4)				

PCR, polymerase chain reaction; GM-EIA, galactomannan enzyme immunoassay; NA, not applicable; − negative; + positive.

**Table 3:** Overview of species detected by culture and *Aspergillus* PCR.

Culture-Positive Species	No.	PCR-Positive Specimens	No.
*A. fumigatus* species complex (s.p)	85	*A. fumigatus* s.c	66
*A. terreus* s.c	53	*A. terreus* s.c	30
*A. flavus* s.c	12	*A. flavus* s.c	6
*A. nidulans* s.c	6	*A. niger* s.c	3
*A. fumigatus* s.c and *A. terreus* s.c	5		
*A. niger* s.c	5		
*A. niger* s.c and *A. flavus* s.c	4		
*A. glaucus* s.c	1		
*A. versicolor* s.c	1		

PCR, polymerase chain reaction.

## Data Availability

The data presented in this study are available in the article.
